# Supplementary material for: Ultrastructure of influenza virus ribonucleoprotein complexes during viral RNA synthesis
Source: Commun Biol. 2021 Jul 9;4:858. doi: 10.1038/s42003-021-02388-4 (PMC8271009; doi:10.1038/s42003-021-02388-4)
Supplement: Supplementary file 2 — Supplementary information [file 42003_2021_2388_MOESM2_ESM.pdf]

Supplementary Information

Ultrastructure of influenza virus ribonucleoprotein complexes during viral RNA synthesis

Masahiro Nakano, Yukihiko Sugita, Noriyuki Kodera, Sho Miyamoto, Yukiko Muramoto, Matthias Wolf, and Takeshi Noda

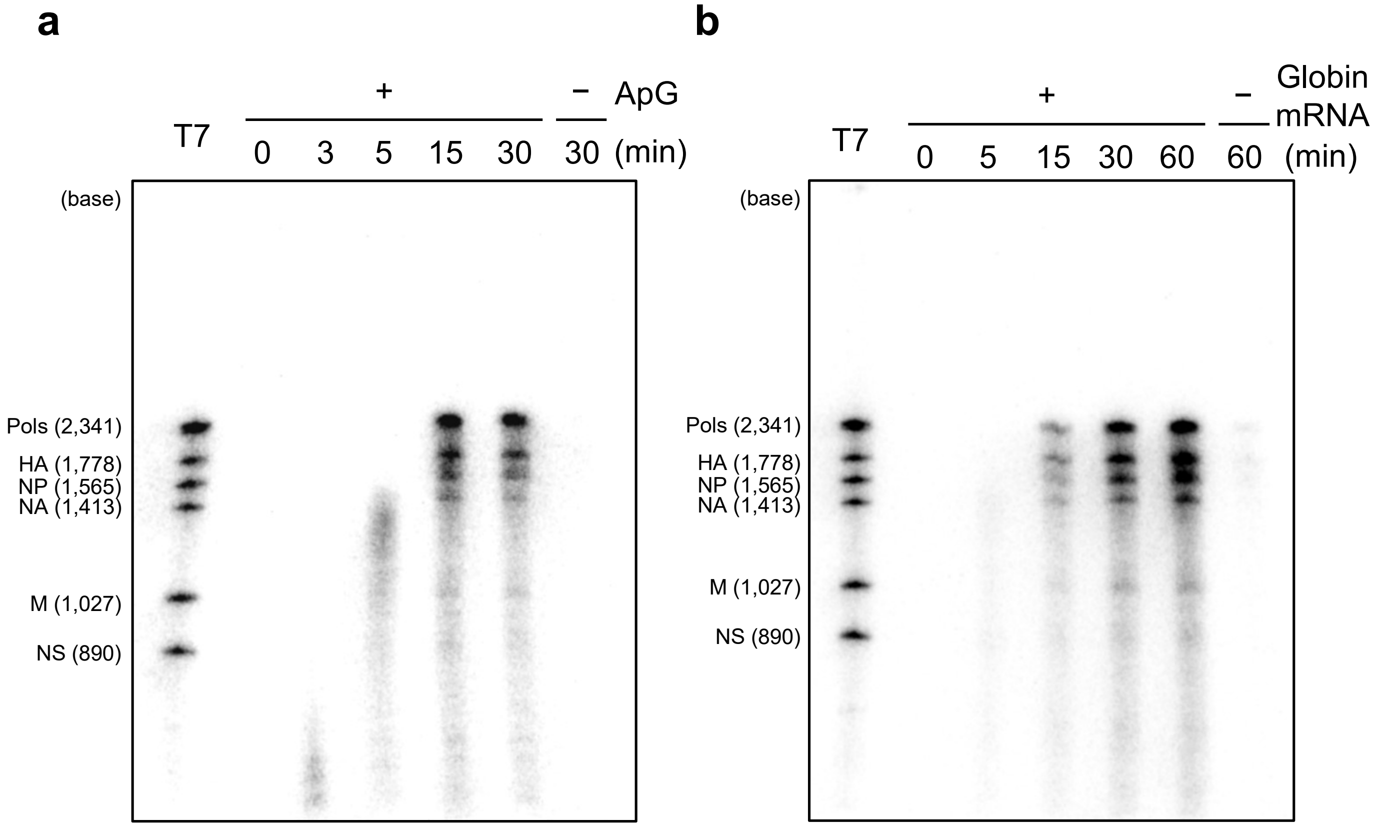

Supplementary Fig. 1. *In vitro* RNA synthesis using virion-derived vRNPs.
a, Time course of *in vitro* RNA synthesis using ApG as a primer. RNA was synthesized *in vitro* with or without ApG for 0, 3, 5, 15, or 30 min. **b,** Time course of *in vitro* RNA synthesis using globin mRNA. RNA was synthesized *in vitro* with or without globin mRNA for 0, 5, 15, 30, or 60 min. Purified RNAs were analysed on a 4% polyacrylamide gel containing 7 M urea and detected by autoradiography. T7 represents a mixture of eight vRNAs synthesized by T7 RNA polymerase, which is the same as used in Fig. 1. Uncropped autoradiograph images are shown in Supplementary Fig. 9.

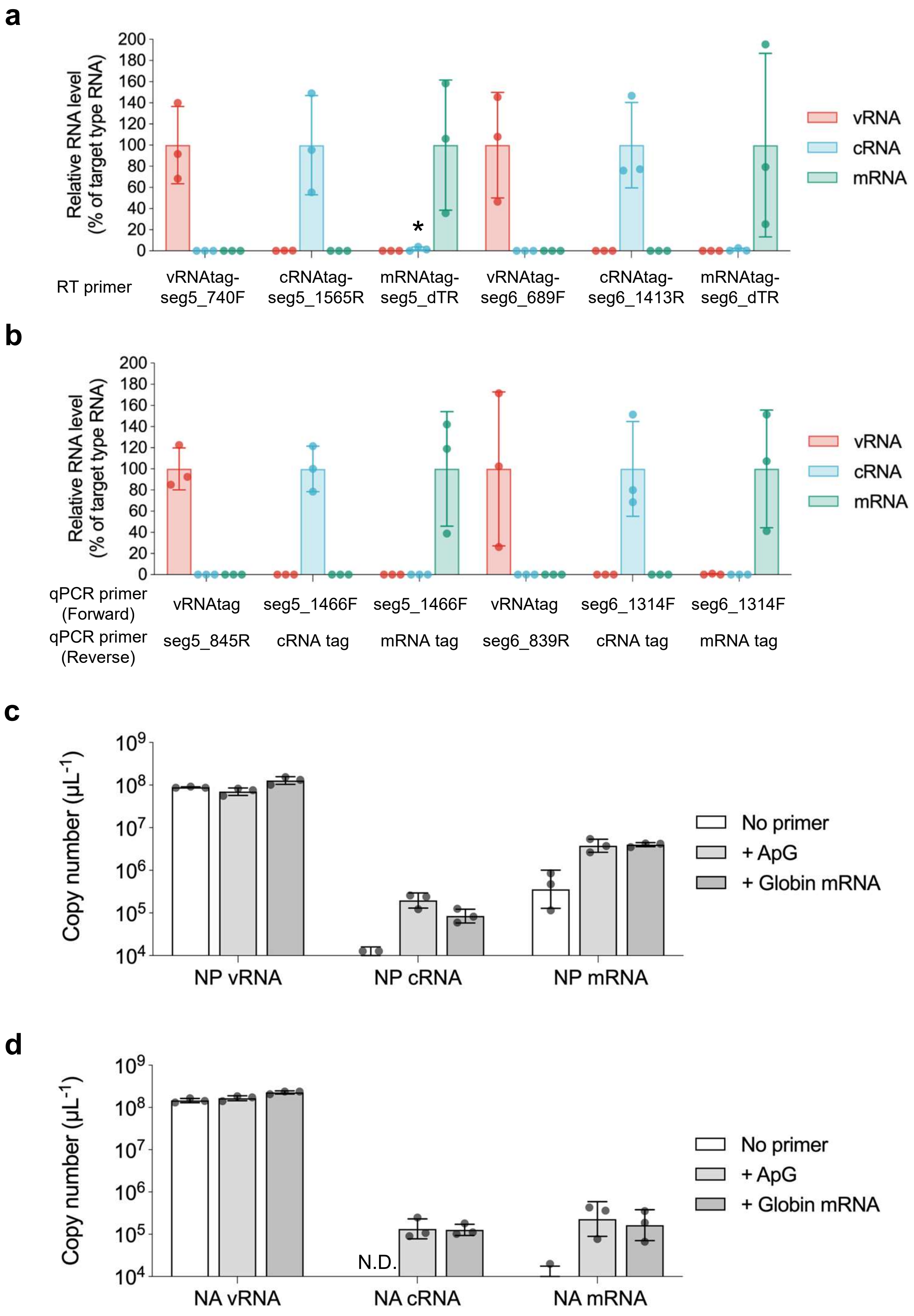
a
Relative RNA level
(% of target type RNA)
200
180
160
140
120
100
80
60
40
20
0
*
vRNA
cRNA
mRNA
RT primer
vRNAtag-seg5_740F
cRNAtag-seg5_1565R
mRNAtag-seg5_dTR
vRNAtag-seg6_689F
cRNAtag-seg6_1413R
mRNAtag-seg6_dTR
b
Relative RNA level
(% of target type RNA)
vRNA
cRNA
mRNA
qPCR primer (Forward)
vRNAtag
seg5_1466F
seg5_1466F
vRNAtag
seg6_1314F
seg6_1314F
qPCR primer (Reverse)
seg5_845R
cRNA tag
mRNA tag
seg6_839R
cRNA tag
mRNA tag
c
Copy number (µL^{-1})
10^9
10^8
10^7
10^6
10^5
10^4
No primer
+ ApG
+ Globin mRNA
NP vRNA
NP cRNA
NP mRNA
d
Copy number (µL^{-1})
No primer
+ ApG
+ Globin mRNA
N.D.
NA vRNA
NA cRNA
NA mRNA

Supplementary Fig. 2. Characterization of RNA products with strand-specific RT-qPCR.

a, Specificity of primers for reverse transcription. First, the standard vRNA (red), cRNA (blue), and mRNA (green) were reverse-transcribed into cDNAs using the tagged RT primers shown in the graph. The obtained tagged cDNAs were then amplified by qPCR using the corresponding tagged portion of the cDNA and a segment-specific oligonucleotide as primers. The average molecular number and standard deviation of triplicate experiments are presented as a percentage of the average value of the target type of RNA. The condition that resulted in the largest non-specific amplification was the case in which the mRNA reverse-transcription primer was used for the cRNA template (asterisk); The relative level of non-specifically amplified RNA was calculated as 1.89% of the mRNA template. **b,** Specificity of primers for qPCR. Standard vRNA (red), cRNA (blue), and mRNA (green) were reverse-transcribed into cDNA using the corresponding RT primers. Using these cDNAs as templates, different sets of primers shown in the graph were used for qPCR. The average molecular number and standard deviation of three independent experiments are presented as in **a**. All estimated values of relative RNA levels were less than 0.5% of each target type RNA, indicating almost no non-specific amplification by different pairs of qPCR primers. **c, d,** Copy numbers of vRNA, cRNA, and mRNA of NP (**c**) or NA (**d**) segment in reaction mixtures were quantified using standard RNAs, which were synthesized with T7 RNA polymerase. N.D. means "not detected" (copy number is less than 1×10^4 μL^{-1}). Data are presented as geometric mean ±S.D. of three independent experiments.

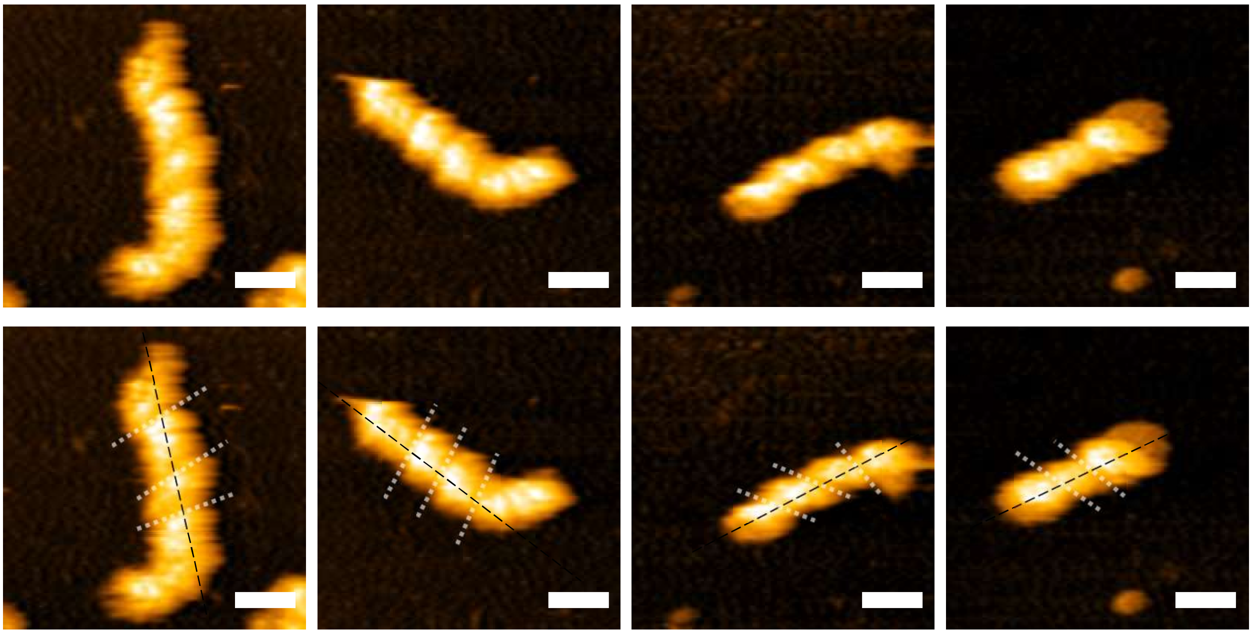

Supplementary Fig. 3. HS-AFM observation of control vRNPs.
In vitro RNA synthesis using virion-derived vRNPs was performed without ApG primer, and samples were observed with HS-AFM (upper panels). For evaluation of the handedness of vRNPs, long axis and helical groove of vRNP are shown in black and white dashed lines, respectively (lower panels). Scale bars, 30 nm.

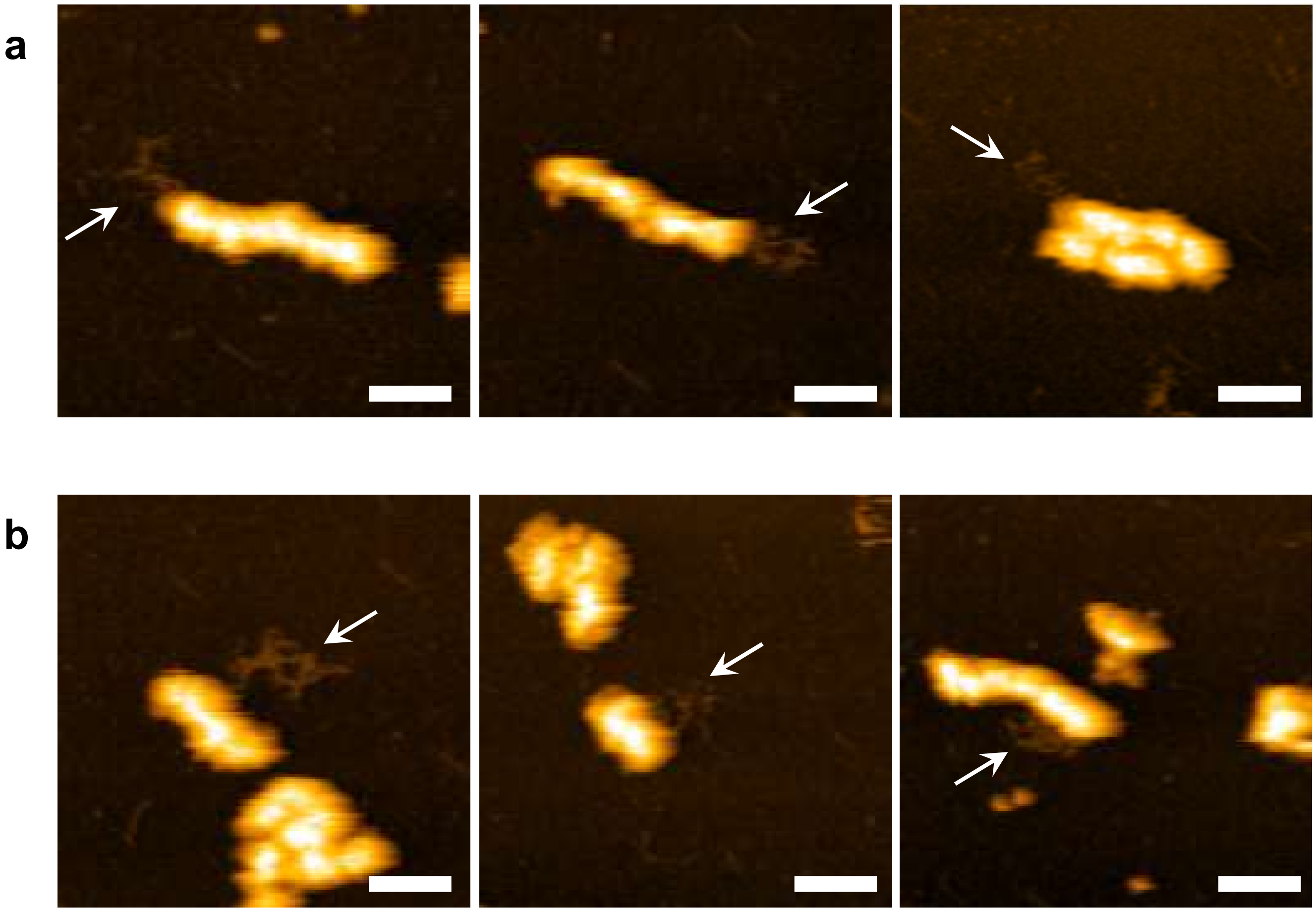

Supplementary Fig. 4. HS-AFM observation of vRNPs associated with a folded RNA.
In vitro RNA synthesis was performed using virion-derived vRNPs and ApG primer, and samples were observed with HS-AFM. Folded RNAs observed at the tip (**a,** arrows) and body of rod-shaped vRNPs (**b,** arrows) are shown. Scale bar on all images represents 50 nm.

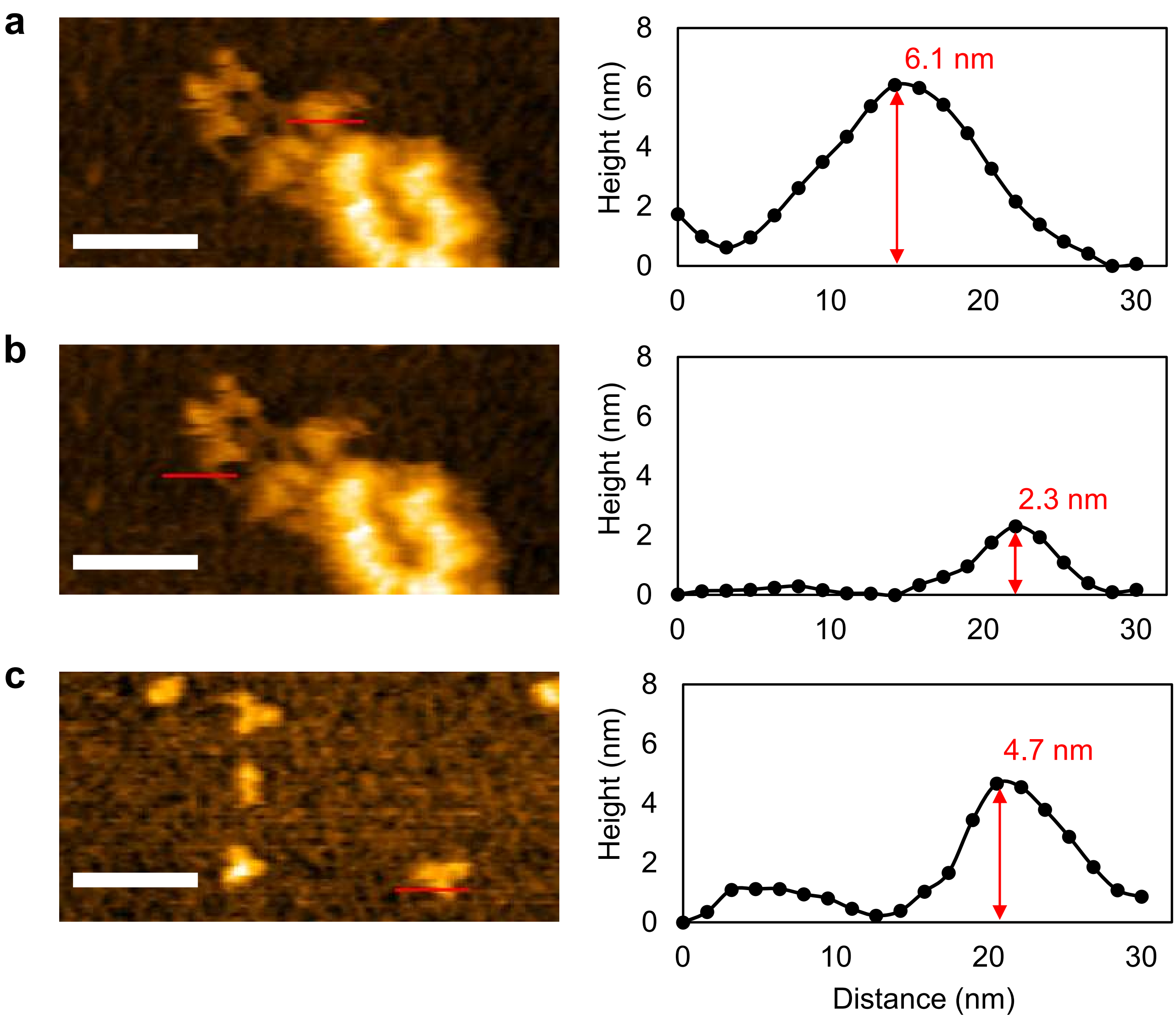

Supplementary Fig. 5. Section analysis of anti-Br-UTP antibody binding to RNA.
a, b, Enlarged HS-AFM images of Fig. 3c are shown on the left. In the right panels, heights of the antibody (**a**) and the folded RNA (**b**) were measured by section analysis at red lines shown in the left panels and revealed to be 6.1 nm and 2.3 nm, respectively. **c,** An HS-AFM image of free anti-Br-UTP antibodies is shown on the left. The section analysis at the red line is shown in the right panel and the height of the antibody was 4.7 nm. Scale bars on all HS-AFM images represent 50 nm.

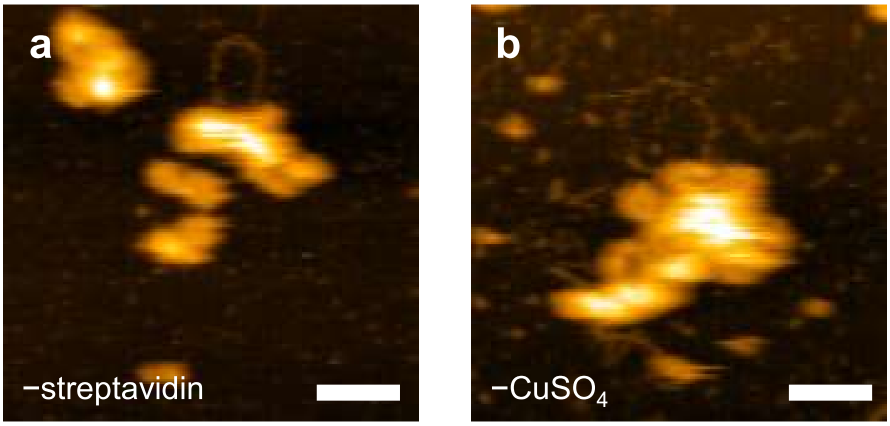

Supplementary Fig. 6. Control experiments for the Click reaction.
The sample for HS-AFM observation was prepared without streptavidin (**a**) or a Click reaction (omitting $CuSO_4$, **b**). No protein binding was observed in any of these images. Results were reproduced at least 5 times. The scale bars represent 50 nm.

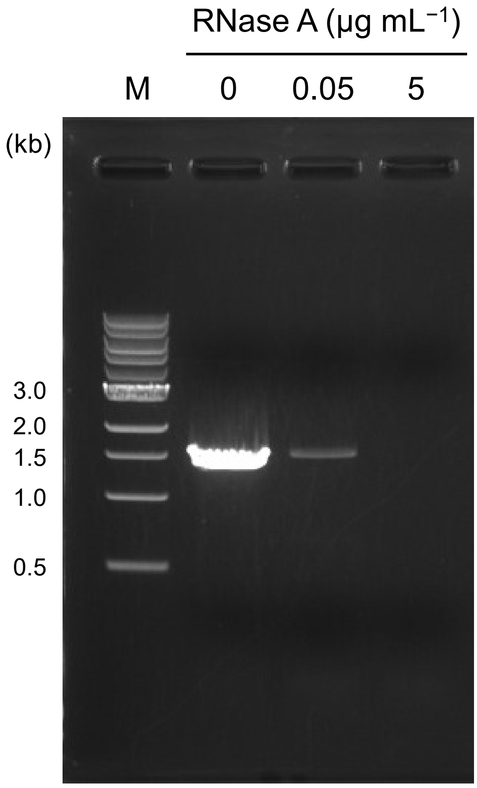

Supplementary Fig. 7. Lack of intact, residential vRNA in RNase A-treated vRNP.
vRNP was treaded with indicated concentrations of RNase A and total RNA was purified. Purified RNA was used in RT-PCR to amplify the DNA fragment of the full-length NP segment (1,565 bp). The PCR product was analysed on 1% agarose gel electrophoresis. M indicates DNA size marker (New England Biolabs). Uncropped gel image is shown in Supplementary Fig. 9.

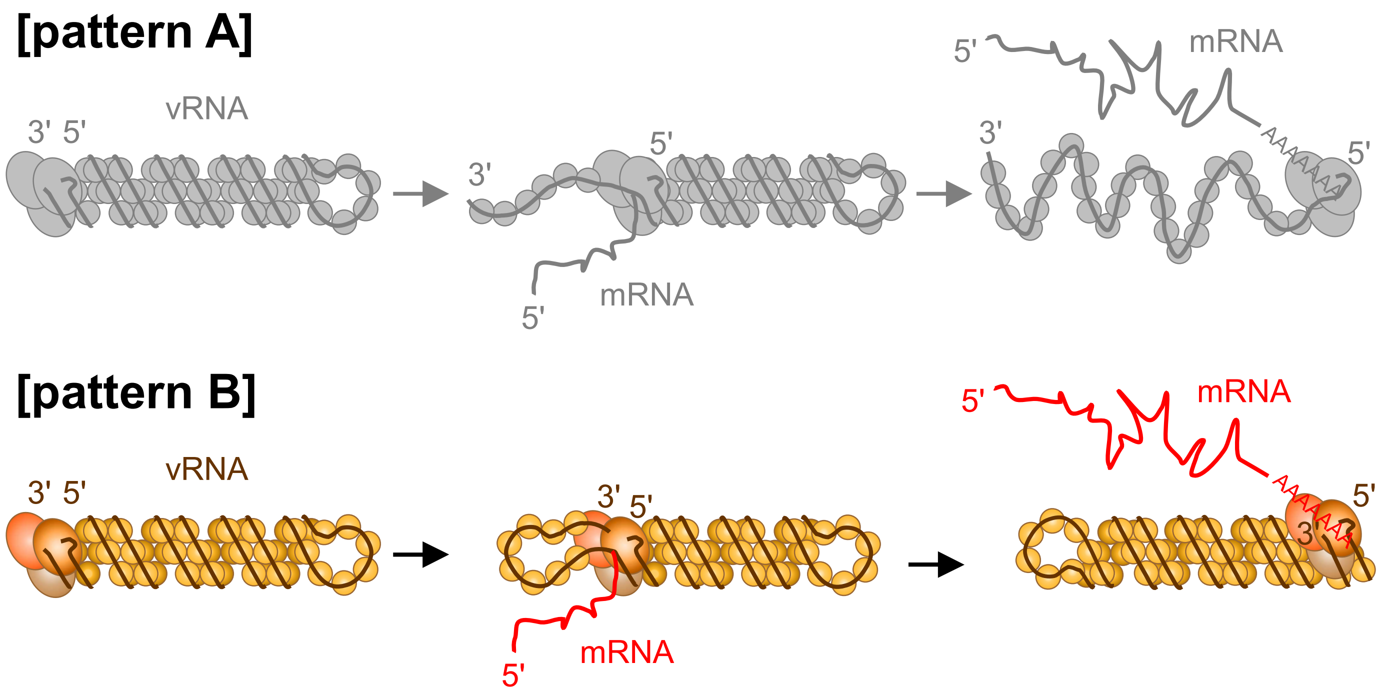

Supplementary Fig. 8. Models for producing folded RNA.
Possible patterns for synthesis of folded RNA are depicted. Patterns A and B show synthesis of mRNA, with the 5' end of vRNA bound to RNA polymerase during transcription. In pattern A, the 3' end of vRNA is detached from the RNA polymerase. In this case, the helical structure of vRNP is relaxed and a single NP-RNA strand appears. In pattern B, both the 5' and 3' ends of vRNA are bound to RNA polymerase. Due to the binding of the 3' end of vRNA to the polymerase, the transcribed vRNA assembles into a helical structure, and the vRNP is able to keep its helical rod shape.

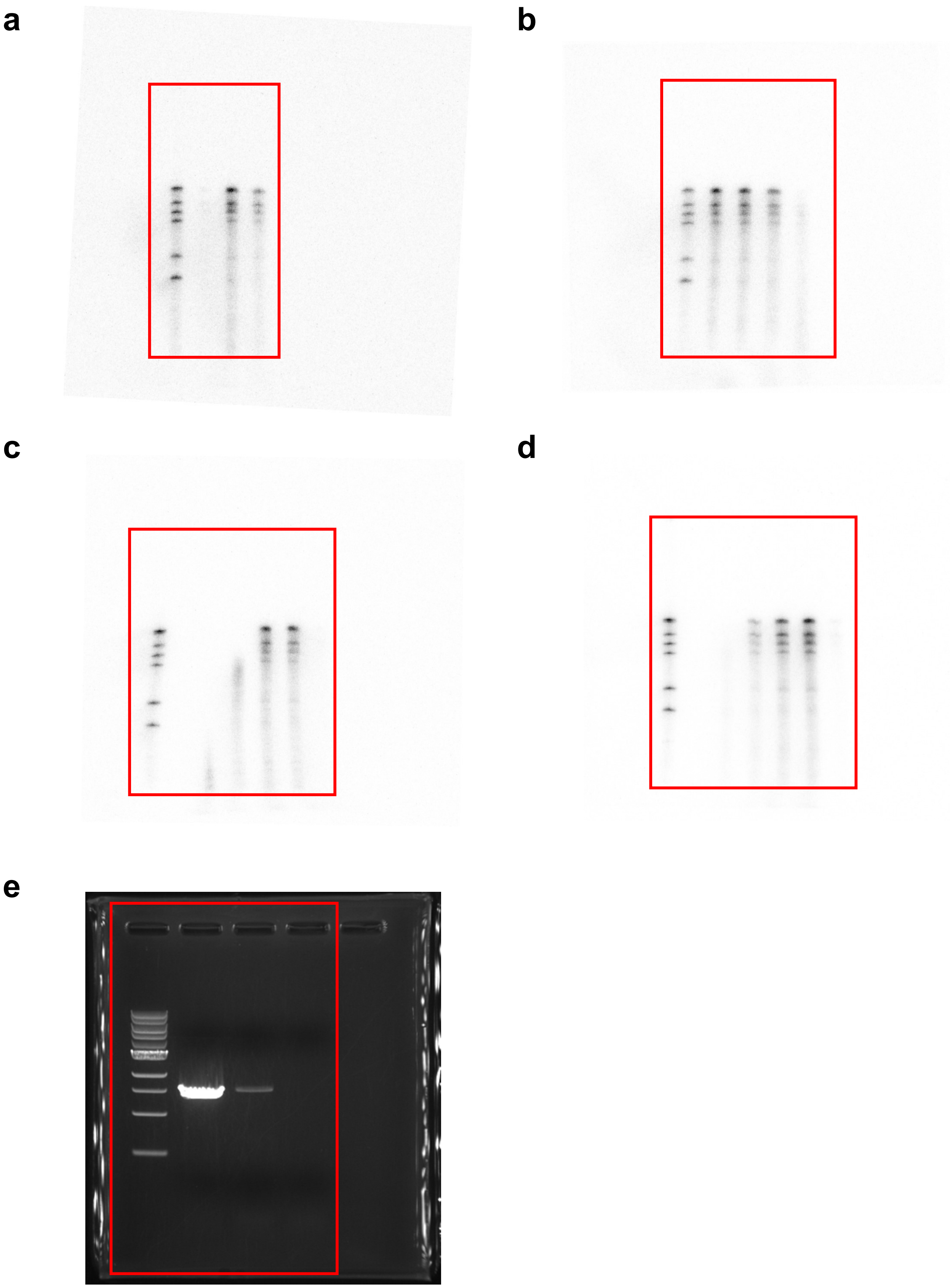

Supplementary Fig. 9. Uncropped autoradiograph and gel images.
a, Uncropped autoradiograph for Fig. 1a. **b,** Uncropped autoradiograph for Fig. 1b. **c,** Uncropped autoradiograph for Supplementary Fig. 1a. **d,** Uncropped autoradiograph for Supplementary Fig. 1b. **e,** Uncropped gel for Supplementary Fig. 7.

Supplementary Table 1. Quantifications of the observed vRNP species.

Primer	Number of vRNPs		
	vRNPs without nascent RNA	vRNPs with folded RNA	vRNPs with looped RNA
None	4,529 (99.04%)	40 (0.87%)	4 (0.09%)
ApG	702 (87.97%)	67 (8.40%)	29 (3.63%)

Supplementary Table 2. Primers used for preparation of standard RNAs.

Target		Primer name	Sequence (5'-3')
NP	vRNA	PR8seg5_1F	AGCAAAAGCAGGGTAGATAATCAC
		T7_PR8seg5_1565R	GGATCCTAATACGACTCACTATAGGGAGTAGAAACAAGGGTATTTTTCTT
	cRNA	T7_PR8seg5_1F	GGATCCTAATACGACTCACTATAGGGAGCAAAAGCAGGGTAGATAATCAC
		PR8seg5_1565R	AGTAGAAACAAGGGTATTTTTCTT
	mRNA	T7_PR8seg5_1F	GGATCCTAATACGACTCACTATAGGGAGCAAAAGCAGGGTAGATAATCAC
		PR8seg5_dTR	TTTTTTTTTTTTTTTTCTTTAATTGTCGTACTCCTC
NA	vRNA	PR8seg6_1F	AGCAAAAGCAGGGGTTTAAAATGA
		T7_PR8seg6_1413R	GGATCCTAATACGACTCACTATAGGGAGTAGAAACAAGGAGTTTTTTGAA
	cRNA	T7_PR8seg6_1F	GGATCCTAATACGACTCACTATAGGGAGCAAAAGCAGGGGTTTAAAATGA
		PR8seg6_1413R	AGTAGAAACAAGGAGTTTTTTGAA
	mRNA	T7_PR8seg6_1F	GGATCCTAATACGACTCACTATAGGGAGCAAAAGCAGGGGTTTAAAATGA
		PR8seg6_dTR	TTTTTTTTTTTTTTTTGAACAGACTACTTGTCAATG

Supplementary Table 3. Primers used for preparation of marker RNAs.

Target	Primer name	Sequence (5'-3')
PB2	WSNseg1_1F	AGCGAAAGCAGGTCAATTATATTC
	T7_WSNseg1_2341R	GGATCCTAATACGACTCACTATAGGGAGTAGAAACAAGGTCGTTTTTAAA
PB1	WSNseg2_1F	AGCGAAAGCAGGCAAACCATTTGA
	T7_WSNseg2_2341R	GGATCCTAATACGACTCACTATAGGGAGTAGAAACAAGGCATTTTTTCAT
PA	WSNseg3_1F	AGCGAAAGCAGGTACTGATTCAAA
	T7_WSNseg3_2233R	GGATCCTAATACGACTCACTATAGGGAGTAGAAACAAGGTACTTTTTTGG
HA	WSNseg4_1F	AGCAAAAGCAGGGGAAAATAAAAA
	T7_WSNseg4_1775R	GGATCCTAATACGACTCACTATAGGGAGTAGAAACAAGGGTGTTTTTCCT
NP	WSNseg5_1F	AGCAAAAGCAGGGTAGATAATCACTC
	T7_WSNseg5_1565R	GGATCCTAATACGACTCACTATAGGGAGTAGAAACAAGGGTATTTTTCTT
NA	WSNseg6_1F	AGCGAAAGCAGGAGTTTAAATGAATCCAAACC
	T7_WSNseg6_1409R	GGATCCTAATACGACTCACTATAGGGAGTAGAAACAAGGAGTTTTTTGAA
M	WSNseg7_1F	AGCAAAAGCAGGTAGATATTGAAA
	T7_WSNseg7_1027R	GGATCCTAATACGACTCACTATAGGGAGTAGAAACAAGGTAGTTTTTTAC
NS	WSNseg8_1F	AGCAAAAGCAGGGTGACAAAGACA
	T7_WSNseg8_890R	GGATCCTAATACGACTCACTATAGGGAGTAGAAACAAGGGTGTTTTTTAT

Supplementary Table 4. Primers used for RT-qPCR.

Target		Purpose	Primer name	Sequence (5'-3')
NP	vRNA	Reverse transcription	vRNAtag-PR8seg5_740F	GGCCGTCATGGTGGCGAATCTGCTGCACAAAAAGCAATGATGG
		qPCR	vRNAtag	GGCCGTCATGGTGGCGAAT
			PR8seg5_845R	CTCAATATGAGTGCAGACCGTGCT
	cRNA	Reverse transcription	cRNAtag-PR8seg5_1565R	GCTAGCTTCAGCTAGGCATCAGTAGAAACAAGGGTATTTTTCTTT
		qPCR	PR8seg5_1466F	CGATCGTGCCTTCCTTTGACATGA
			cRNAtag	GCTAGCTTCAGCTAGGCATC
	mRNA	Reverse transcription	mRNAtag-PR8seg5_dTR	GCCAGATCGTTCGAGTCGTTTTTTTTTTTTTTTTTCTTTAATTG
		qPCR	PR8seg5_1466F	CGATCGTGCCTTCCTTTGACATGA
			mRNAtag	GCCAGATCGTTCGAGTCGT
NA	vRNA	Reverse transcription	vRNAtag-PR8seg6_689F	GGCCGTCATGGTGGCGAATTTTTACTATAATGACTGATGGCCCG
		qPCR	vRNAtag	GGCCGTCATGGTGGCGAAT
			PR8seg6_839R	CACTTTGCCGGTATCAGGGTAACA
	cRNA	Reverse transcription	cRNAtag-PR8seg6_1413R	GCTAGCTTCAGCTAGGCATCAGTAGAAACAAGGAGTTTTTTGAAC
		qPCR	PR8seg6_1314F	GGCGTGAATAGTGATACTGTAGAT
			cRNAtag	GCTAGCTTCAGCTAGGCATC
	mRNA	Reverse transcription	mRNAtag-PR8seg6_dTR	GCCAGATCGTTCGAGTCGTTTTTTTTTTTTTTTTTGAACAGACT
		qPCR	PR8seg6_1314F	GGCGTGAATAGTGATACTGTAGAT
			mRNAtag	GCCAGATCGTTCGAGTCGT
