# Supplementary material for: Ultrastructure of influenza virus ribonucleoprotein complexes during viral RNA synthesis
Source: Commun Biol. 2021 Jul 9;4:858. doi: 10.1038/s42003-021-02388-4 (PMC8271009; doi:10.1038/s42003-021-02388-4)
Supplement: Supplementary file 3 — Description of Additional Supplementary Files [file 42003_2021_2388_MOESM3_ESM.pdf]

Description of Additional Supplementary Files

File Name: Supplementary Data 1
Description: Source data for Fig.6b and Supplementary Fig. 2.

File Name: Supplementary Movie 1
Description: **Digestion of looped RNA with RNase A**
During HS-AFM observation of looped RNA associated with vRNP, RNase A was added to the liquid chamber at a final concentration of 0.5 µg mL−1. Scan area: 300×300 nm2. Observation period: 120 sec.

File Name: Supplementary Movie 2
Description: **Digestion of looped RNA with RNase III**
RNase III was added at a final concentration of 0.02 U µL−1 during HS-AFM observation of looped RNA. Scan area: 300×300 nm2. Observation period: 76 sec.

File Name: Supplementary Movie 3
Description: **Binding of antidsRNA antibodies to looped RNA**
Binding of anti-dsRNA antibodies to looped Page 15 of 16 RNA was observed by HS-AFM. Scan area: 300×300 nm2. Observation period: 9 sec.

File Name: Supplementary Movie 4
Description: **Binding of antidsRNA antibodies to structured RNA**
Binding of anti-dsRNA antibodies to structured RNA was observed by HS-AFM. Scan area: 300×300 nm2. Observation period: 9 sec.
