# Supplementary material for: Ultrastructure of influenza virus ribonucleoprotein complexes during viral RNA synthesis
Source: Commun Biol. 2021 Jul 9;4:858. doi: 10.1038/s42003-021-02388-4 (PMC8271009; doi:10.1038/s42003-021-02388-4)
Supplement: Supplementary file 9 — Reporting Summary [file 42003_2021_2388_MOESM9_ESM.pdf]

Corresponding author(s): Takeshi Noda
Last updated by author(s): Feb 17, 2021

Reporting Summary

Nature Research wishes to improve the reproducibility of the work that we publish. This form provides structure for consistency and transparency in reporting. For further information on Nature Research policies, see our Editorial Policies and the Editorial Policy Checklist.

Statistics

For all statistical analyses, confirm that the following items are present in the figure legend, table legend, main text, or Methods section.

n/a	Confirmed	
☐	☒	The exact sample size (n) for each experimental group/condition, given as a discrete number and unit of measurement
☐	☒	A statement on whether measurements were taken from distinct samples or whether the same sample was measured repeatedly
☐	☒	The statistical test(s) used AND whether they are one- or two-sided<br>*Only common tests should be described solely by name; describe more complex techniques in the Methods section.*
☒	☐	A description of all covariates tested
☐	☒	A description of any assumptions or corrections, such as tests of normality and adjustment for multiple comparisons
☐	☒	A full description of the statistical parameters including central tendency (e.g. means) or other basic estimates (e.g. regression coefficient) AND variation (e.g. standard deviation) or associated estimates of uncertainty (e.g. confidence intervals)
☐	☒	For null hypothesis testing, the test statistic (e.g. F, t, r) with confidence intervals, effect sizes, degrees of freedom and P value noted<br>*Give P values as exact values whenever suitable.*
☒	☐	For Bayesian analysis, information on the choice of priors and Markov chain Monte Carlo settings
☒	☐	For hierarchical and complex designs, identification of the appropriate level for tests and full reporting of outcomes
☒	☐	Estimates of effect sizes (e.g. Cohen's d, Pearson's r), indicating how they were calculated

Our web collection on statistics for biologists contains articles on many of the points above.

Software and code

Policy information about availability of computer code

Data collection	HS-AFM images were captured using Igor Pro v.6.3.4.1. with Eagle package v.2.5.5. Fluorescent microscope images were collected using DeltaVision SoftWoRx v.6.5.2. Cryo-EM data were collected automatically using the open source software LEGINON.
Data analysis	Statistical analyses were performed by using available R packages (https://www.r-project.org/). HS-AFM images were treated with the lab-made software Kodec for noise reduction and flattening. Cryo-EM data processing was performed with the open source software IMOD 4.11 and commercially available AMIRA. Image J was used to contrast all images.

For manuscripts utilizing custom algorithms or software that are central to the research but not yet described in published literature, software must be made available to editors and reviewers. We strongly encourage code deposition in a community repository (e.g. GitHub). See the Nature Research guidelines for submitting code & software for further information.

Data

Policy information about availability of data

All manuscripts must include a data availability statement. This statement should provide the following information, where applicable:

- Accession codes, unique identifiers, or web links for publicly available datasets
- A list of figures that have associated raw data
- A description of any restrictions on data availability

All data generated during the current study are available from the corresponding author on reasonable request.

Field-specific reporting

Please select the one below that is the best fit for your research. If you are not sure, read the appropriate sections before making your selection.

[x] Life sciences [ ] Behavioural & social sciences [ ] Ecological, evolutionary & environmental sciences

For a reference copy of the document with all sections, see nature.com/documents/nr-reporting-summary-flat.pdf

Life sciences study design

All studies must disclose on these points even when the disclosure is negative.

Sample size	No sample-size calculation was performed.
Data exclusions	No data was excluded from analysis.
Replication	All attempts at replication were successful.
Randomization	No randomization was performed.
Blinding	No blinding was carried out.

Reporting for specific materials, systems and methods

We require information from authors about some types of materials, experimental systems and methods used in many studies. Here, indicate whether each material, system or method listed is relevant to your study. If you are not sure if a list item applies to your research, read the appropriate section before selecting a response.

Materials & experimental systems

n/a	Involved in the study	
[ ]	[x]	Antibodies
[ ]	[x]	Eukaryotic cell lines
[x]	[ ]	Palaeontology and archaeology
[x]	[ ]	Animals and other organisms
[x]	[ ]	Human research participants
[x]	[ ]	Clinical data
[x]	[ ]	Dual use research of concern

Methods

n/a	Involved in the study	
[x]	[ ]	ChIP-seq
[x]	[ ]	Flow cytometry
[x]	[ ]	MRI-based neuroimaging

Antibodies

Antibodies used	A rabbit polyclonal antibody for influenza A virus nucleoprotein (NP) was obtained from GeneTex (GTX125989) and used at a 1:1000 dilution. Anti-dsRNA mouse monoclonal antibody J2 was obtained from Scicons (10010200) and used at a 1:500 dilution. Goat Alexa Fluoro 488-conjugated anti-mouse antibody (A11001, Thermo Fisher Scientific) and goat Alexa Fluoro 555-conjugated anti-rabbit antibody (A21428, Thermo Fisher Scientific) were used at 1:2000 and 1:1000 dilutions, respectively.
Validation	All antibodies are commercially available. See the corresponding webpages for reference and validation.

Eukaryotic cell lines

Policy information about cell lines

Cell line source(s)	Vero cells (CCL-81) were obtained from ATCC.
Authentication	The cell line was provided by supplier (ATCC).
Mycoplasma contamination	The elimination of mycoplasma was confirmed by ATCC.
Commonly misidentified lines (See ICLAC register)	No commonly misidentified cell lines were used.
